# Editorial: Extracellular Vesicles in Infectious Diseases

**DOI:** 10.3389/fcimb.2021.697919

**Published:** 2021-07-01

**Authors:** Neta Regev-Rudzki, Shulamit Michaeli, Ana Claudia Torrecilhas

**Affiliations:** ^1^ Department of Biomolecular Sciences, Weizmann Institute of Science, Rehovot, Israel; ^2^ Bar-Ilan University, Ramat Gan, Israel; ^3^ Department of Pharmacy, Federal University of São Paulo (UNIFESP), São Paulo, Brazil

**Keywords:** extracellular vesicles, pathogens, virus, parasites, fungi, infectious disease

## 

Extracellular vesicles (EVs) isolated from pathogens mediate communication between parasites and their hosts under a variety of physiological and pathological conditions. EVs deliver cell-free messages *via* a transfer of RNA, proteins, and even DNA to modulate and induce inflammation and to control the host infection process. EVs can provide valuable information on how a pathogen sends messages to other pathogens and hosts (Torrecilhas et al., 2012; Campos et al., 2015; Ofir-Birin and Regev-Rudzki, 2019; Torrecilhas et al. 2020). This Research Topic provides an overview of the mechanism of EV-mediated communication between hosts and viruses, parasites, and fungi. This Research Topic consists of 17 papers, including 8 reviews and 9 original papers. Several studies on this topic assessed the effects of EVs on the interactions between pathogens and hosts, and the mechanisms of EV-mediated communication between hosts and pathogens were also addressed.

The first original article published in this Frontiers in Cellular and Infection Microbiology Parasite and Host issue is from Duguet’s group (Duguet et al.). They showed that EVs isolated from *Caenorhabditis elegans* contain microRNAs and small regulators that affect biological processes and comment on their role in host-nematode communication. Parasite-derived miRNAs regulate host immune system mRNAs.

The paper by Zhang et al. discusses how *Echinococcus granulosus* protoscoleces (PSCs) and hydatid cysts release EVs that are exosome-like based on size and morphology. The authors analyzed the miRNA, circRNA, and lncRNA profiles of the 20 most abundant miRNAs in EVs and explored their possible roles in biological processes, especially in pathways associated with pathogenicity and the host immune response. These EVs contain small RNA species, including specific miRNAs that are homologous to host miRNAs and induce immunomodulation in the host.

Another article discusses the role of *Cryptococcus deneoformans*, a fungus that causes meningoencephalitis in immunocompromised patients. In this study, the authors showed that fungal shedding of EV RNAs and cryptococcal intersectin protein (Cin1) govern a unique Cin1-Wsp1-Cdc42 endocytic pathway required for intracellular transport, virulence, and regulated particle secretion. Cin1 has clinical importance as an exRNA, and studies using cutting-edge technology in cryptococcal pathogenesis may contribute to the discovery of novel therapeutic strategies (Liu et al.).

The study by Nawaz et al. included proteomic analysis of EVs purified from the saliva of *Haemaphysalis longicornis*. The bush tick is a vector of human disease-causing agents, such as the agent causing thrombocytopenia syndrome. The authors found that EVs carry proteins that may be helpful during tick development and vesicular proteins involved in proton transport, detoxification, ECM-receptor interaction, ribosomes, RNA transport, ABC transporters, and oxidative phosphorylation.

Several studies on this topic have assessed the role of EV release in leishmaniasis. Saini and Rai showed that treatment with linoleic acid (LA) inhibited *Leishmania donovani* infection in macrophages, suppressed the parasitic load, and reduced the levels of IL-12 and *i*NOS. LA promoted a protective-type immune response in the infected host and inhibited the release of *Leishmania*-derived microvesicles. Soares’ team also presented data on macrophage activation using EVs isolated from *L. infantum L. braziliensis*, and *L. amazonensis*. EVs from *L. braziliensis* and *L. infantum* parasites failed to induce a proinflammatory response. EVs from both the *L. infantum WT* and LPG-deficient mutant (LPG-KO) did not show any differences in their interactions with macrophages, suggesting that LPG alone was not sufficient for activation (Nogueira et al.). Another group investigated whether *Leishmania amazonensis* or EVs modulate B-1 cell activation and differentiation. *L. amazonensis* infection in B-1 cells decreased the production of NO and ROS. TLR-2, TLR-6 and TLR-9 had significantly higher expression in B-1 cells from mice intraperitoneally injected or stimulated with EVs (Reis et al.).

Gualdrón-López et al. discussed multiparameter flow cytometry combined with cell and EV purification techniques to determine and investigate the interactions of plasma-derived EVs from human spleen cells from *Plasmodium vivax*-infected patients (PvEVs). They used size-exclusion chromatography (SEC) to separate EVs from the bulk of the soluble plasma proteins and stained isolated EVs with fluorescent lipophilic dyes (Gualdrón-López et al.).

This Research Topic also includes reviews that discuss the role of EVs in viral infections. Dr. Soekmadji’s group discussed the role of EVs in hepatic fibrosis and how understanding their biological mechanism of action might be beneficial for developing therapeutic strategies to treat chronic liver disease (Lim et al.). Another review discussed EVs containing infective viral genomes that are secreted into the extracellular space (Martins and Alves). EVs can trigger antiviral responses and cytokine secretion even in uninfected cells near the infection, and the role of EVs during viral infections is crucial for comprehending viral mechanisms and how to respond to emerging viral diseases (Martins and Alves). In this review, the symbiosis between the TV-specific endosymbiont viruses *Trichomonasvirus* and *Trichomonas vaginalis* was studied by exploiting sEVs as a vehicle for intercellular communications and by modifying their protein cargo to suppress host immune activation, including NF-κB activation and increases in IL-8 and RANTES. The virus may offer evolutionary benefits to its protozoan host, at least partially, by altering the immunomodulatory properties of EVs spreading from the site of infection to noninfected immune effector cells. EVs serve as vehicles for intercellular communication and modify their protein cargo to suppress host immune activation (Govender et al.).

A review on influenza virus infection discusses the EVs released from infected cells, implying the functional relevance of EVs for influenza virus dissemination, EV-based influenza vaccines, and therapeutic strategies to combat the influenza virus (Jiang et al.).

Another review shows how cross-kingdom sRNA trafficking occurs in EVs participating in sRNA delivery in the host during infection, as well as the production of EVs by bacterial and fungal pathogens that help establish the disease. Bacterial vesicle RNA cargo plays roles in recipient host cells by regulating gene expression and modulating the immune response. In fungi, the RNA molecules present in EVs are diverse and participate in communication between the host and pathogenic fungi (Munhoz da Rocha et al.). In parallel, other authors discussed the diverse functions of EVs in fungi, from the regulation of physiological events and responses to specific environmental conditions to the mediation of highly complex interkingdom communications and the function of EVs as vehicles for the delivery of biologically active molecules (Rizzo et al.). Protozoan EV reviews showed that the EVs isolated from pathogenic protozoa are disseminated alongside their biomolecules, and there are specific immune cell responses to protozoan parasite-derived EVs. Protozoan-host interactions release EVs that are crucial for the immunomodulatory events triggered by parasites (Torrecilhas et al.). Additionally, EVs from these organisms have a role in the digestive tracts of invertebrate hosts prior to parasite transmission. This review summarizes the available data on how EVs from medically important trypanosomatids affect parasite interactions with vertebrate and invertebrate hosts (Torrecilhas et al.; Olajide and Cai). Schistosomes release EVs that modulate the host immune response and EV-harbored miRNAs to upregulate the innate immune response of the M1 pathway and downregulate differentiation towards adaptive Th2 immunity. EVs may facilitate the development of novel tools for diagnostics and deliver therapy in relation to schistosomiasis, as well as to immune-associated disorders (Avni and Avni).

Overall, this Research Topic, “Extracellular Vesicles in Infectious Diseases” bring together important original papers and reviews to understand the interactions between pathogens and hosts and deliver scientific reports on the mechanisms of EV-mediated communication between hosts and pathogens (**Figure 1**).

**Figure 1:**
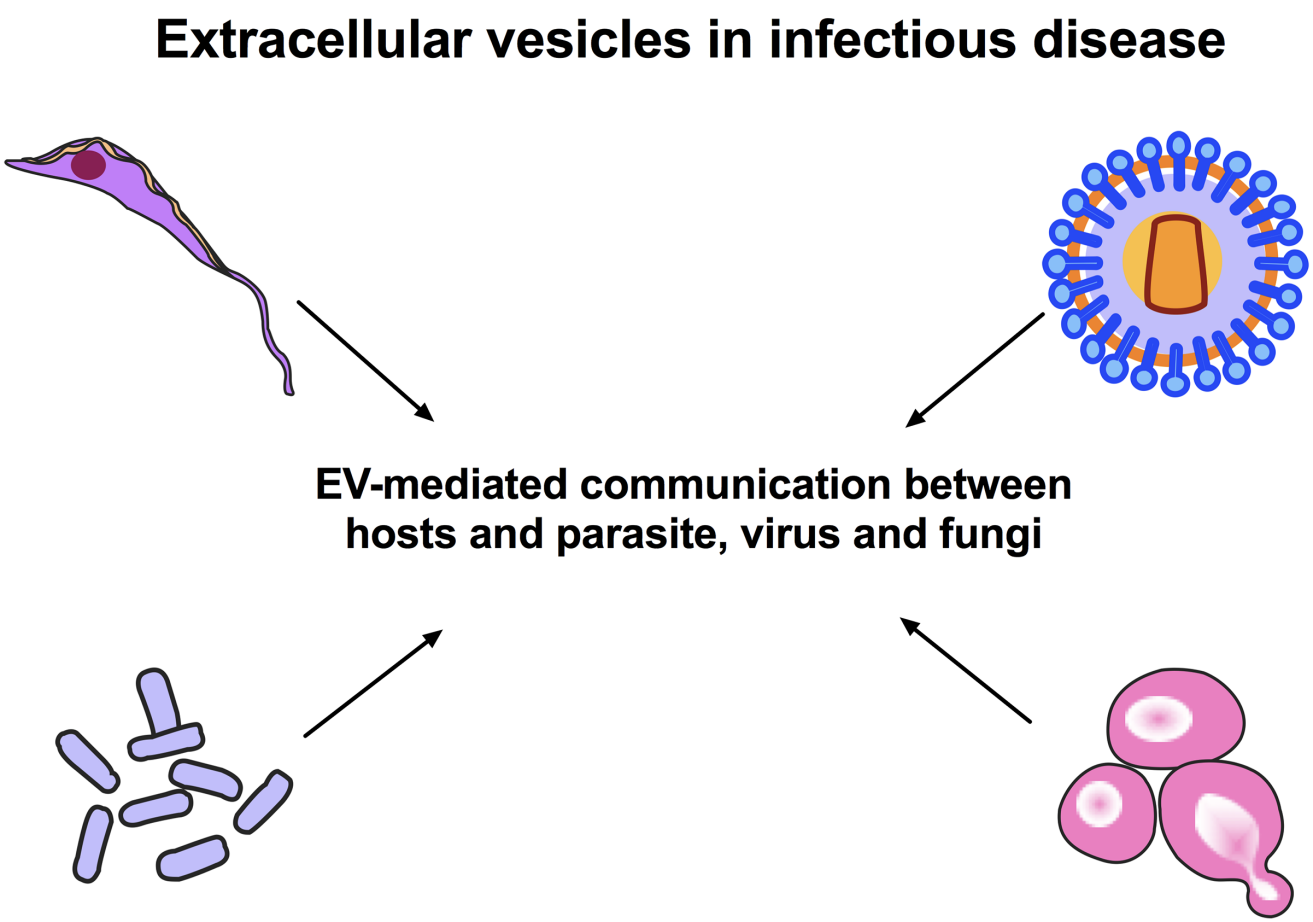
Extracellular Vesicles in Infectious Diseases. EVs isolated from parasites, fungi and viruses are known to mediate communication under a variety of physiological and pathological conditions and modulate the host immune system. EVs are a new research area that can provide valuable information on how a pathogen sends messages to other pathogens and hosts. The fundamental point for the control of endemic parasitic diseases is an understanding of the mechanisms involved in the pathogen-host interactions.

## Author Contributions

MS prepared the draft. NR-R, SM and ACT revised the draft and approved the final version of the draft. NR-R, SM and ACT assisted in submission. All authors contributed to the article and approved the submitted version.

## Funding

Fundação de Amparo à Pesquisa do Estado de São Paulo (FAPESP) number 2019/15909-0 and Conselho Nacional de Desenvolvimento Científico e Tecnológico (CNPq) grant 408186/2018-6 and FAPESP 2020/07870-4 (ACT).

## Conflict of Interest

The authors declare that the research was conducted in the absence of any commercial or financial relationships that could be construed as a potential conflict of interest.
